# Artificial intelligence and multimodal diagnostic approaches in cardiovascular disease

**DOI:** 10.47487/apcyccv.v6i4.532

**Published:** 2025-12-29

**Authors:** Fernando A. Ramos-Zaga

**Affiliations:** 1 Universidad Privada del Norte, Lima, Perú. Universidad Privada del Norte Universidad Privada del Norte Lima Peru

**Keywords:** Artificial Intelligence, Machine Learning, Diagnostic Techniques, Cardiovascular, Precision Medicine, Inteligencia Artificial, Aprendizaje Automático, Técnicas de Diagnóstico Cardiovascular, Medicina de Precisión

## Abstract

**Objective.:**

Evaluate the impact and clinical applicability of artificial intelligence (AI) models in cardiovascular diagnosis, assessing their potential to improve diagnostic accuracy, operational efficiency, and reliability compared with conventional methods.

**Materials and:**

**Methods.** A critical review of the recent literature was conducted, encompassing retrospective studies, multicenter trials, and external validations that employed machine learning and deep learning algorithms applied to imaging modalities, electrocardiographic and phonocardiographic signals, as well as clinical and proteomic biomarkers.

**Results.:**

Evidence indicates that in cardiac imaging, automated segmentation and ventricular dysfunction detection achieved accuracy metrics exceeding 90%, suggesting readiness for clinical integration. In cardiac signals, deep learning models demonstrated area under the ROC curve values of approximately 0.99 for predicting atrial fibrillation and ischemic heart disease, further supported by explainability techniques. Regarding biomarkers, ensemble models achieved diagnostic accuracies above 95%, and the integration of proteomic and clinical data substantially enhanced predictive performance. Nonetheless, decreased performance in external validations, limited generalizability to heterogeneous populations, and clinicians’ reluctance due to insufficient explainability remain major barriers.

**Conclusion.:**

Artificial intelligence in cardiovascular diagnostics holds transformative potential by improving accuracy, reducing interobserver variability, and expanding access in resource-limited settings. However, its consolidation into routine practice requires robust multicenter validations, seamless interoperability with clinical workflows, and strengthened explainability, prerequisites for incorporation into clinical guidelines and precision medicine strategies.

## RESUMEN

**Objetivo.:**

Evaluar el impacto y la aplicabilidad clínica de los modelos de inteligencia artificial (IA) en el diagnóstico cardiovascular, analizando su capacidad para mejorar la precisión, la eficiencia operativa y la confiabilidad en comparación con métodos convencionales.

**Materiales y:**

**métodos.** Se realizó una revisión crítica de la literatura reciente, incluyendo estudios retrospectivos, multicéntricos y validaciones externas que aplicaron algoritmos de *machine learning* y *deep learning* a modalidades de imagen, señales electrocardiográficas y fonocardiográficas, así como biomarcadores clínicos y proteómicos.

**Resultados.:**

Los hallazgos muestran que en imágenes cardíacas, la segmentación automática y la detección de disfunción ventricular alcanzan métricas de precisión superiores al 90%, con potencial de integración clínica. En señales cardíacas, el *deep learning* ha demostrado áreas bajo la curva ROC cercanas a 0,99 en la predicción de fibrilación auricular y cardiopatía isquémica, reforzadas por técnicas explicables. En biomarcadores, los modelos de *ensemble* superaron el 95% de precisión diagnóstica y la integración de proteómica con datos clínicos incrementó la capacidad predictiva. Sin embargo, se observó una disminución en el desempeño de validaciones externas, limitaciones en la generalización a poblaciones heterogéneas y reticencia por la escasa explicabilidad de algunos algoritmos.

**Conclusión.:**

La IA aplicada al diagnóstico cardiovascular exhibe un potencial transformador al mejorar la precisión diagnóstica, reducir la variabilidad y ampliar el acceso a entornos con recursos limitados. No obstante, su consolidación en la práctica clínica requiere validaciones multicéntricas, interoperabilidad con flujos clínicos reales y fortalecimiento de la explicabilidad, condiciones esenciales para su inclusión en guías de práctica clínica y medicina de precisión.

## Introduction

Cardiovascular diagnostics are undergoing a transitional phase driven by the integration of artificial intelligence (AI) tools across multiple levels of clinical practice. The convergence of advances in imaging modalities, cardiac signal analysis, and biomarker technologies has created unprecedented opportunities to enhance diagnostic accuracy, efficiency, and scalability ^1^. This evolution, however, is not occurring in isolation: it is shaped by the rising global prevalence of cardiovascular diseases and the imperative to optimise resources within increasingly strained health systems.

Interest in applying algorithms to the medical field dates back to the earliest expert systems ^2^, later evolving into machine learning methods and, more recently, deep learning approaches applied to imaging and physiological signals ^3^. In cardiology, these strategies have enabled notable advances, including the automated detection of ventricular dysfunction ^4^, the prediction of atrial fibrillation from sinus-rhythm electrocardiograms ^5^, and the integration of proteomic biomarkers with large-scale clinical datasets ^6^.

Nonetheless, a persistent gap remains between the technical performance of these models and their clinical maturity. Despite outstanding metrics in controlled research settings, multicentre studies have shown substantial declines in performance when algorithms are applied to heterogeneous populations or to imaging and signal data of variable quality ^7^. This issue, described in the computer science literature as a generalisation challenge ^8^, is particularly salient in cardiology, where the wide diversity of phenotypes and comorbidities demands methodological robustness and flexibility.

The rationale for advancing this field lies in the fact that current developments have yet to translate into routine clinical practice. Outstanding performance metrics reported in retrospective studies contrast with the limited availability of longitudinal, external, and multicentre validations needed to demonstrate true clinical utility ^9^. In addition, the lack of sufficient interpretability reinforces clinicians’ reluctance to adopt “black-box” algorithms, hindering their integration into decision-making processes.

The practical implications of this evolution are considerable. An AI system capable of robustly interpreting images, electrocardiograms, and biomarkers could not only optimise diagnostic processes in specialised settings, but also expand access in resource-limited contexts through portable devices and automated analyses ^10^. Likewise, the integration of multimodal information would enable clinicians to address clinical complexity from a more holistic perspective, overcoming the constraints of unimodal approaches ^11^.

The issues outlined above intersect with broader contemporary challenges in medicine, including the need to develop ethical and transparent models, to prevent algorithmic bias, and to ensure that technological innovations do not exacerbate existing structural inequalities ^12^. They also align with the priorities of precision medicine, which aims to integrate molecular, clinical, and population-level data to individualise care ^13^. Thus, the debate surrounding the maturity of AI in cardiology extends beyond technical considerations and moves into spheres of social, ethical, and economic relevance.

Within this context, the aim of this work is to critically examine recent advances in AI-assisted cardiovascular diagnostics, encompassing imaging modalities, cardiac signals, and biomarkers, in order to establish a reference framework for assessing their maturity, accuracy, and clinical applicability across diverse settings. The contribution of this article lies in providing a synthesis that highlights both current opportunities and limitations, thereby paving the way for a more robust, interpretable, and equitable integration of AI into cardiovascular clinical practice.

## Materials and methods

This article adopts a critical narrative review format following the SANRA (Scale for the Assessment of Narrative Review Articles) framework, as its primary aim is to integrate and interpret recent evidence on AI in cardiovascular diagnostics, with particular attention to its clinical applicability and degree of technological maturity. This approach enables the findings to be contextualised within a broader theoretical and clinical framework, moving beyond the simple aggregation of quantitative results and offering a more conceptual understanding of the phenomenon.

The methodological purpose was to identify, examine, and critically discuss recent advances in the use of AI for cardiovascular diagnosis through imaging, signal analysis, and biomarkers. The review aims to assess the robustness, interpretability, and clinical applicability of these models, highlighting both their achievements and their limitations in medical practice.

We included original studies (prospective, retrospective, multicentre, or clinical) that met the following criteria: a) application of AI models to cardiovascular diagnosis using imaging, signal analysis, or biomarkers; b) reporting of quantitative performance metrics (area under the curve [AUC], sensitivity, specificity, F1-score, or others); and c) availability of internal or external validation. Narrative reviews, editorials, conference abstracts, conceptual studies without empirical data, and articles lacking diagnostic metrics or a validated clinical application were excluded.

The literature search was conducted between January and August 2025. Combinations of controlled and uncontrolled terms were applied using Boolean operators: (“artificial intelligence” OR “machine learning” OR “deep learning”) AND (“cardiovascular diagnosis” OR “cardiac imaging” OR “electrocardiogram” OR “biomarkers”). Articles published between 2018 and 2025 in English or Spanish were included to capture the most influential contemporary literature.

The initial search across Scopus, PubMed, and IEEE Xplore identified a total of 152 records, distributed as follows: IEEE Xplore (66), Scopus (49), and PubMed (37). This breadth reflects the growing body of research on AI applied to cardiovascular diagnosis, spanning medical imaging models to predictive algorithms based on signals and biomarkers. The substantial number of redundant entries and thematically peripheral publications aligns with patterns reported in other emerging areas of computational medicine.

Automatic removal of duplicates using Zotero resulted in the exclusion of 24 records, leaving 128 unique articles for the initial screening phase. This step reduced potential bias due to bibliographic repetition and ensured the uniqueness of each reference assessed. During title and abstract screening, 26 records were excluded because they did not meet the eligibility criteria. The main reasons for exclusion were the absence of direct application to cardiovascular diagnosis, a purely technical orientation without clinical validation, or a lack of alignment with the study’s objective.

**Figure 1:**
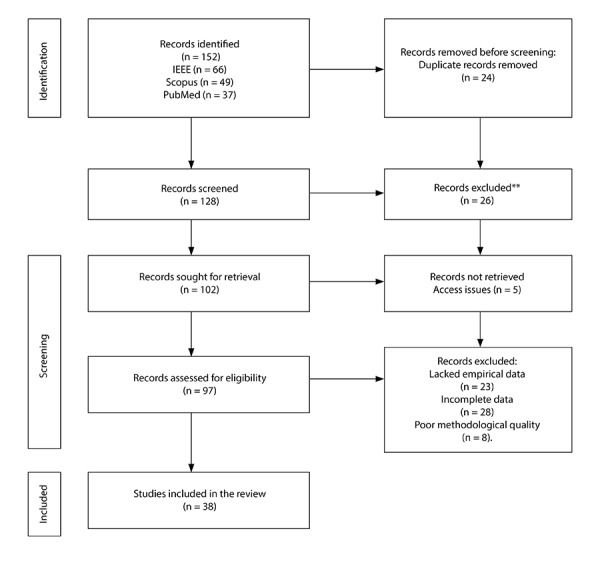
Identification of studies through databases and records.

Full-text retrieval was attempted for 102 studies, of which five could not be obtained due to access restrictions or editorial availability. The remaining 97 articles were assessed for eligibility using predefined criteria related to methodological quality, diagnostic relevance, and reporting of performance metrics. In this phase, 59 records were excluded either for lack of empirical data (n = 23), incomplete information (n = 28), or insufficient methodological quality (n = 8). Ultimately, 38 studies met all inclusion criteria (Figure 1).

Search results were refined through a structured review process. A standardised data extraction form was used to collect information on the type of AI model, diagnostic modality, sample size, study design, performance metrics, and validation approach. Bibliographic records were organised and cross-checked using Zotero, ensuring traceability and duplicate control.

The information was organised according to the three principal diagnostic dimensions: cardiovascular imaging, cardiac signals, and biomarkers. A qualitative critical analysis was undertaken to identify patterns in model performance, methodological consistency, and degree of clinical maturity. The synthesis aimed to integrate the evidence through an interpretative lens, assessing model interpretability, interoperability, and generalisability beyond their technical metrics.

## Results

### Imaging-based diagnosis

Advances in cardiovascular imaging have progressively enhanced the precision and efficiency of clinical evaluation, particularly in echocardiography, magnetic resonance imaging (MRI), computed tomography (CT), and multimodal approaches. In echocardiography, improvements in automatic segmentation of cardiac chambers and valves have surpassed traditional performance benchmarks, demonstrating notable robustness to anatomical variability and image quality. A U-Net-based model incorporating ASPP modules achieved an F1-score of 0.91 and a Dice coefficient of 0.9284, suggesting that these tools may be approaching readiness for clinical integration ^14^. In an analysis of 15,000 studies, automated segmentation of the left ventricle enabled highly accurate estimation of the ejection fraction (EF), with a 93% success rate in view identification, although performance declined in the presence of atrial fibrillation and low-quality images ^15^.

Complementary developments include an adversarial model that achieved Dice coefficients above 86% across multiple cardiac chambers, with a volume correlation of 0.94 relative to manual segmentation ^16^. Other approaches, such as trilateral attention networks, have demonstrated real-time segmentation and quantification capabilities, outperforming expert readers across four independent datasets ^17^. Additionally, the incorporation of automated quality-control systems increased the proportion of usable frames to 96%, further strengthening the feasibility of clinical implementation ^18^.

Automated detection of ventricular dysfunction has also achieved noteworthy progress. A multicentre study involving more than 147,000 patients reported an AUC of 0.94 for reduced left ventricular EF and 0.84 for right ventricular dysfunction, with low absolute error compared with reference standards ^19^. Another analysis of over 200,000 individuals showed that electrocardiography (ECG) can predict filling pressures and grades of left ventricular diastolic dysfunction with AUCs exceeding 0.91, comparable to echocardiographic performance ^20^. In a more accessible setting, the combination of phonocardiography and ECG using wearable patches achieved an AUROC of up to 0.91, with sensitivities above 90%, underscoring its utility in resource-limited environments ^21^. Multimodal integration of auscultation and ECG provided further benefit, yielding AUCs of 0.75 in both internal and external cohorts, confirming that combining sensors can enhance the detection of ventricular dysfunction ^22^.

MRI and CT have provided new avenues for detecting and quantifying cardiovascular disease. In ischaemic heart disease, deep neural networks optimised using the Levenberg-Marquardt algorithm achieved an accuracy of 86.39% and an AUC of 0.93 for myocardial ischaemia detection, with good correlation in ventricular volume estimation ^23^. Complementarily, AI-assisted coronary CT angiography demonstrated a sensitivity of 75% and a specificity of 70% compared with invasive angiography, with a particularly high negative predictive value in women and performance surpassing that of single-photon emission computed tomography (SPECT) ^24^. For fibrosis and viability assessment, a native CT-based algorithm showed significant correlation with MRI late-gadolinium enhancement (r = 0.77-0.81), with near-perfect reproducibility, although validated in a relatively small cohort ^25^. Meanwhile, cine-MRI analysis reached validation accuracies of 89%, but performance dropped to 70% in external testing, underscoring the need for further optimisation ^26^. Other methods, such as support vector machines applied to post-contrast MRI, achieved accuracies of 71% and sensitivities of 72%, outperforming deep networks in some scenarios ^27^. Texture analysis in echocardiography yielded agreement rates of up to 76% compared with MRI, with improved performance in transmural scarring and after contrast administration, suggesting potential utility in post-infarction assessment ^28^.

Integration of imaging modalities with additional sources of information has emerged as a particularly promising strategy. Fusion of coronary CT angiography and MRI using an XGBoost model achieved an AUC of 0.86, with external validations reporting values up to 0.92, outperforming traditional clinical cardiovascular risk scores ^11^. DenseResNet architectures applied to combined MRI, CT, and echocardiography yielded an accuracy of 98.4%, with both sensitivity and specificity exceeding 97%, clearly surpassing unimodal models ^29^. In a more modest approach, late fusion of ECG data and clinical records achieved an accuracy of 72.2%, though its performance was constrained by limited sample size ^30^. Finally, the integration of echocardiography, ECG, and biochemical parameters reached an accuracy of 89.87%, with a recall of 91.20% and an F1-score of 89.13%, findings validated in clinical cohorts that support the real-world applicability of such multimodal algorithms ^31^ (Table 1).

**Table 1:** AI models applied to cardiovascular imaging diagnosis

Study group	Predominant models	Modality / data type	Learning	Validation strategy
Echocardiographic segmentation and quantification ^(14-18)^	CNN (U-Net, attention-based variants, and GAN)	2D echocardiography images	Supervised and semi-supervised	Cross-validation and external validation across multiple datasets
Ventricular dysfunction detection and functional prediction ^(19-22)^	Deep CNN and multimodal models (ECG + phonocardiogram)	2D images and 1D signals	Supervised	Multicentre and external validations
Structural diagnosis using MRI and CT ^(23-28)^	CNN, SVM, and ensemble algorithms	2D and 3D images	Supervised	Internal and external validation
Multimodal integration ^(11, 29-31)^	DenseResNet, XGBoost, and ensemble methods	Combined MRI, CT, echocardiography, ECG, and biomarkers	Supervised	External and clinical validations

CNN: convolutional neural networks. SVM: support vector machine. MRI: magnetic resonance imaging. CT: cardiac computed tomography. ECG: electrocardiogram.

### Signal-based diagnosis

Advances in cardiac signal analysis have opened an increasingly broad landscape for the non-invasive diagnosis of diverse cardiovascular conditions. In the field of ECG analysis, AI models have shown remarkable capability for the early detection of atrial fibrillation. A retrospective study including more than 135,000 ECG recordings reported that both classical algorithms and deep learning models achieved sensitivities of 90%; however, specificity was higher with deep learning, reaching 69% compared with 62% for conventional models, using cardiologist interpretation as the reference standard ^32^.

The development of predictive models has further expanded the scope of these technologies. In a cohort of more than 318,000 patients and over half a million ECGs, a deep learning model trained to predict paroxysmal atrial fibrillation in individuals with normal sinus rhythm achieved an AUROC of 0.905 ± 0.007 for one-month prediction, incorporating explainable techniques that enabled interpretation of the contributions of different waveform segments ^33^.

Other approaches have focused on integrating classical ECG parameters, such as P-wave morphology and heart rate variability. Using this strategy, an ensemble learning model achieved an accuracy of 92%, sensitivity of 88%, and specificity of 96%, with an AUROC of 0.911 in public datasets, demonstrating that stacking can outperform other combinatory methods ^34^. In a different line of work, training convolutional neural networks on tens of thousands of Holter segments yielded sensitivities of 97.1% and specificities of 94.5%, with an AUROC close to 0.99. The use of Grad-CAM enabled validation of the clinical coherence of the regions highlighted by the model, supporting the robustness of this approach in real-world settings ^35^.

The application of ECG analysis to the detection of ischaemic heart disease has shown consistent results across multiple settings. In one of the most widely used datasets for this purpose, a support vector machine (SVM) model achieved an accuracy of 97.98% in classifying ischaemic segments, demonstrating its utility for large-scale analysis, although clinical cohort validation is still needed ^36^. In a study combining ECG and vectorcardiography (VCG), accuracy reached 90.3%, with equivalent sensitivities and specificities and an AUC of 0.814 in external cohorts, confirming adequate generalisability ^37^. Multicentre validation in 595 patients further supported the applicability of machine learning-assisted VCG, with sensitivities exceeding 97% in men and 90% in women, and consistent cross-validation against coronary angiography ^38^.

Phonocardiogram analysis has likewise shown substantial progress in the identification of valvular heart disease. In a multicentre study involving nearly 500 patients, sensitivities ranged from 71.4% to 100% and specificities from 83.5% to 100%, with optimal performance for mitral stenosis, where 100% was achieved across all metrics, validated prospectively against echocardiography ^39^. The incorporation of mobile-device signals and self-supervised learning techniques increased accuracy to above 99.4%, even under noisy conditions, opening the door to large-scale screening in mobile health settings ^40^. Across model architectures, Vision Transformer-based approaches achieved an accuracy of 99.90% and an F1-score of 99.95%, outperforming more traditional methods and highlighting the advantages of attention mechanisms ^41^. In a real-world clinical scenario, an AI-enabled digital stethoscope achieved a sensitivity of 94.1%, markedly higher than the 41.2% obtained by primary care physicians, albeit with lower specificity, reinforcing its potential role in early detection and selective referral ^42^.

Analysis of cardiac murmurs with direct correlation to echocardiography has demonstrated utility in both paediatric and adult populations. In a prospective cohort of 116 children, classical models achieved accuracies above 90% for distinguishing organic murmurs associated with congenital heart disease, with direct validation against echocardiography ^43^. In a more advanced framework, hierarchical multitask models trained on public datasets enabled not only murmur detection and grading but also risk estimation, with interpretability provided through SHAP (Shapley additive explanations), making them a potentially valuable tool for optimising referral pathways ^44^. Finally, the use of mel-spectrograms processed through transfer-learning networks yielded rapid and robust classifications of murmur presence and severity, with explainability supported by Occlusion Sensitivity, demonstrating their value as a complementary screening method to echocardiography ^45^ (Table 2).

**Table 2:** AI models applied to cardiac signal-based diagnosis

Study group	Predominant models	Modality / data type	Learning	Validation strategy
Atrial fibrillation detection and prediction ^(32-35)^	CNN, recurrent neural networks (RNN/LSTM), and ensemble learning	ECG and Holter recordings (1D signals)	Supervised and partially explainable	Internal and external validations, including some multicentre studies
Ischemic heart disease diagnosis ^(36-38)^	SVM, CNN, and hybrid ECG-VCG models	ECG and vectorcardiography	Supervised	Cross-validation and multicentre validation against coronary angiography
Valvular disease identification using phonocardiograms ^(39-42)^	CNN, Vision Transformer, and self-supervised models	Digital phonocardiograms	Supervised and self-supervised	Prospective and real-world validation
Advanced murmur analysis and hierarchical classification ^(43-45)^	CNN with transfer learning, multitask models, and SHAP	Mel-spectrograms and phonocardiograms	Supervised with interpretability	Internal and external validations using echocardiography as reference

CNN: convolutional neural networks. RNN: recurrent neural network. LSTM: long short-term memory. SVM: support vector machine. ECG: electrocardiogram. VCG: vectorcardiogram.

### Biomarker-based diagnosis

The study of biomarkers in cardiovascular diagnosis has evolved toward the integration of machine learning methods capable of handling large volumes of clinical and laboratory data. In a comparative analysis including eight classical and ensemble algorithms, extensive preprocessing, comprising normalisation, balancing, and variable selection, enabled accuracies exceeding 98%, confirming the value of combining multiple techniques in large and heterogeneous cohorts ^46^.

In a smaller cohort of 224 patients, evaluation of six different algorithms demonstrated the feasibility of using machine learning for preventive diagnosis based on laboratory data. Although detailed numerical metrics were not reported, the findings suggest that this approach may hold clinical utility; however, the absence of extensive validation limits its standardisation in routine practice ^47^.

The potential of ensemble models was also evident in an analysis of 100 patients, where five algorithms were applied to basic clinical variables such as age, blood pressure, and cholesterol levels. Gradient Boosting achieved the highest accuracy at 92.5%, outperforming Random Forest and other approaches. However, the small sample size limits the generalisability of these findings and underscores the need for studies with greater statistical power ^48^.

Another study explored dimensionality reduction applied to 303 records comprising 13 clinical variables, optimising classification performance across several models. The k-nearest neighbours (KNN) algorithm demonstrated superior performance, with accuracies of 83.8% in training and 80% in testing, supported by 10-fold cross-validation. These findings position KNN as a useful tool in clinical settings working with structured data, although there remains room for improvement compared with more complex algorithms ^49^.

Integration of proteomic biomarkers with clinical data has broadened the landscape for cardiovascular risk prediction. In an analysis of the UK Biobank incorporating blood-based proteomic profiles and clinical variables, the use of an Explainable Boosting Machine yielded an AUROC of 0.767 and an AUPRC of 0.2405 when relying solely on proteomic data. The addition of clinical information improved these metrics to 0.785 and 0.2835, respectively, outperforming traditional models as well as machine learning algorithms such as LightGBM ^6^^)^ (Table 3).

**Table 3:** AI models applied to cardiovascular biomarker-based diagnosis

Study group	Predominant models	Modality / data type	Learning	Validation strategy
Classical and ensemble models using clinical and laboratory data ^(46,47)^	Decision trees, Random Forest, Gradient Boosting, and combined models	Structured clinical variables	Supervised	Internal cross-validation
Algorithms in small cohorts and structured datasets ^(48,49)^	Gradient Boosting, Random Forest, and KNN	Basic clinical data (age, blood pressure, cholesterol)	Supervised	10-fold cross-validation
Large-scale proteomic and clinical integration ^(6)^	Explainable Boosting Machine and LightGBM	Proteomic + clinical data	Supervised with interpretability	Validation in a population biobank (UK Biobank)

KNN: k-nearest neighbours. LightGBM: Light Gradient Boosting Machine. BP: blood pressure. UK: United Kingdom.

## Discussion

Advances in AI applied to cardiovascular diagnosis are demonstrating increasing clinical impact, albeit with nuances regarding their applicability and reliability. In echocardiography, automatic chamber segmentation has achieved precision metrics that consistently surpass manual practice, with Dice coefficients approaching 0.93 and the ability to automatically compute EF in large cohorts. These findings suggest utility in routine clinical practice, particularly for reducing interobserver variability, although limitations persist in patients with atrial fibrillation or low-quality images ^14^^,^^15^.

Automated detection of ventricular dysfunction using non-invasive data has demonstrated AUC values exceeding 0.90 in populations of more than 100,000 patients, representing a substantial advance in diagnostic scalability. Its greatest strength lies in its capacity for population-level screening, although specificity in clinically complex subgroups may limit applicability for individual decision-making ^19^^,^^20^.

In MRI and CT, deep learning algorithms and kernel-based methods have achieved accuracies above 85% for detecting ischaemia and quantifying fibrosis. However, performance drops in external validations and heterogeneity in sample sizes highlight the need for multicentre validation protocols before clinical adoption. Their main contribution lies in enhancing objectivity and reproducibility, although their reliability still depends on methodological standardisation ^23^^,^^26^.

Multimodal approaches have been the most consistent in terms of clinical impact. Fusion of imaging modalities with clinical data has yielded AUC values approaching 0.92, significantly outperforming conventional risk scores and confirming added value for prognostic stratification. The ability to integrate diverse sources of information marks a step toward decision-support models with genuine applicability in hospital settings ^11^^,^^29^.

Signal analysis has shown outstanding performance in atrial fibrillation, with AUROC values nearing 0.99 in Holter recordings and explainability consistent with clinical criteria, supporting its reliability and opening opportunities for early detection in primary care. Nonetheless, generalisation across different devices and clinical contexts remains a challenge ^33^^,^^35^. In ischaemic heart disease, ECG- and VCG-based models achieved accuracies above 90% with validation against coronary angiography, demonstrating applicability in screening contexts, although translation into routine clinical practice will require prospective validation ^37^^,^^38^.

AI-assisted phonocardiography has surpassed the diagnostic accuracy of general practitioners in the assessment of valvular heart disease, achieving sensitivities above 94%. Its reliability is strengthened by prospective validations against echocardiography, positioning these tools as valuable complements in primary care and mobile-health (mHealth) environments, with substantial potential for large-scale screening ^39^^,^^42^.

In the field of biomarkers, machine learning models applied to laboratory data achieved accuracies close to 98% in large cohorts, with consistent cross-validation results supporting their value in preventive contexts. Nonetheless, small sample sizes in some studies limit the reliability of certain findings ^46^^,^^48^. The integration of proteomic data with clinical variables in population-based cohorts improved cardiovascular risk prediction and enabled interpretable identification of candidate biomarkers, reinforcing their applicability in personalised medicine and primary prevention strategies ^6^.

The application of AI to cardiovascular diagnosis represents a significant step toward more precise, accessible, and efficient medicine, although persistent challenges continue to shape its clinical implementation. The medical implications of this technology include the potential to standardise diagnostic interpretation, optimise early disease detection, and reduce interobserver variability. However, its real impact will depend on multicentre validations capable of ensuring reproducibility across heterogeneous populations and diverse care settings.

On the other hand, the lack of explainability in some models continues to limit their acceptance among healthcare professionals, as it hinders clinical interpretation and shared decision-making. Moreover, evidence gaps remain regarding longitudinal performance, interoperability with clinical systems, and evaluation of hard outcomes. Overcoming these limitations will require prospective studies, algorithmic transparency, and ethical and regulatory integration that support the responsible use of AI in cardiovascular practice.

Given the narrative nature of this article, several methodological limitations arise, including potential publication bias and heterogeneity among the included studies. No meta-analysis or formal risk-of-bias assessment was conducted; therefore, the findings should be interpreted as a critical synthesis of the available evidence. These constraints limit quantitative inference, although they allow the identification of key trends and evidence gaps relevant for future clinical research.

In conclusion, performance metrics demonstrate transformative potential in terms of scalability, diagnostic precision, and efficiency. However, full clinical applicability depends on multicentre validation, interoperability with real-world workflows, and evidence of impact on clinical outcomes. The reliability of these models is strengthened by explainability and consistent performance in external cohorts, both essential conditions for their integration into clinical practice guidelines.

According to the findings, AI applied to cardiovascular diagnosis shows substantial clinical potential, but its full adoption requires rigorous multicentre validations that confirm the reproducibility of results across diverse populations and real-world care settings. Likewise, model explainability is an essential requirement for strengthening clinical trust, facilitating interpretation of algorithmic decisions, and ensuring ethical and safe integration into medical practice. The future advancement of the field will depend on a balanced combination of technical performance, transparency, and robust clinical evidence.

## References

[1] Topol EJ (2019). High-performance medicine the convergence of human and artificial intelligence. Nat Med.

[2] Shortliffe EH, Buchanan BG (1975). A model of inexact reasoning in medicine Math. Biosci.

[3] Esteva A, Kuprel B, Novoa RA, Ko J, Swetter SM, Blau HM (2017). Dermatologist-level classification of skin cancer with deep neural networks. Nature.

[4] Yao X, Rushlow DR, Inselman JW, McCoy RG, Thacher TD, Behnken EM (2021). Artificial intelligence-enabled electrocardiograms for identification of patients with low ejection fraction a pragmatic, randomized clinical trial. Nat Med.

[5] Kwon D, Kang H, Lee D, Kim YC (2025). Deep learning-based prediction of atrial fibrillation from polar transformed time-frequency electrocardiogram. PLoS ONE.

[6] Climente-González H, Oh M, Chajewska U, Hosseini R, Mukherjee S, Gan W (2025). Interpretable machine learning leverages proteomics to improve cardiovascular disease risk prediction and biomarker identification. Commun Med.

[7] Yu AC, Mohajer B, Eng J (2022). External Validation of Deep Learning Algorithms for Radiologic Diagnosis A Systematic Review. Radiol Artif Intell.

[8] Dietterich T (1995). Overfitting and undercomputing in machine learning. ACM Comput Surv.

[9] Rajkomar A, Dean J, Kohane I (2019). Machine Learning in Medicine. N Engl J Med.

[10] Berkebile JA, Inan OT, Beach PA (2025). Wearable multimodal sensing for quantifying the cardiovascular autonomic effects of levodopa in parkinsonism. Front Netw Physiol.

[11] Pezel T, Toupin S, Bousson V, Hamzi K, Hovasse T, Lefevre T (2025). A Machine Learning Model Using Cardiac CT and MRI Data Predicts Cardiovascular Events in Obstructive Coronary Artery Disease. Radiology.

[12] Obermeyer Z, Emanuel EJ (2016). Predicting the Future - Big Data, Machine Learning, and Clinical Medicine. N Engl J Med.

[13] Collins FS, Varmus H (2015). A New Initiative on Precision Medicine. N Engl J Med.

[14] Lal S (2024). TC-SegNet robust deep learning network for fully automatic two-chamber segmentation of two-dimensional echocardiography. Multimed Tools Appl.

[15] Morales-Galan A, Lopez-Gutierrez P, Garrido-Oliver J, Dux-Santoy L, Majul H, Rivas Catoni L (2025). Artificial intelligence for automatic echocardiography image view detection and left ventricle segmentation and ejection fraction prediction Eur Heart J Cardiovasc. Imaging.

[16] Arafati A, Morisawa D, Avendi MR, Amini MR, Assadi RA, Jafarkhani H (2020). Generalizable fully automated multi-label segmentation of four-chamber view echocardiograms based on deep convolutional adversarial networks. J R Soc Interface.

[17] Zamzmi G, Rajaraman S, Hsu LY, Sachdev V, Antani S (2022). Real-time echocardiography image analysis and quantification of cardiac indices. Med Image Anal.

[18] Geven BWM, Zhao D, Creamer SA, Dillon JR, Quill GM, Edwards NC, Camara O, Puyol-Antón E, Sermesant M, Suinesiaputra A, Tao Q, Wang C (2024). Statistical Atlases and Computational Models of the Heart Regular and CMRxRecon Challenge Papers [Internet].

[19] Vaid A, Johnson KW, Badgeley MA, Somani SS, Bicak M, Landi I (2022). Using Deep-Learning Algorithms to Simultaneously Identify Right and Left Ventricular Dysfunction From the Electrocardiogram. JACC Cardiovasc Imaging.

[20] Lee E, Ito S, Miranda WR, Lopez-Jimenez F, Kane GC, Asirvatham SJ (2024). Artificial intelligence-enabled ECG for left ventricular diastolic function and filling pressure. npj Digit Med.

[21] Zhang WL, Song B, Huang QJ, Quan WW, Zhang RY (2024). Artificial intelligence for automated left ventricular systolic dysfunction detection using a wearable cardiac patch incorporated with synchronized phonocardiogram and electrocardiogram. Eur Heart J.

[22] Shiraga T, Makimoto H, Kohlmann B, Magnisali CE, Imai Y, Itani Y (2023). Improving Valvular Pathologies and Ventricular Dysfunction Diagnostic Efficiency Using Combined Auscultation and Electrocardiography Data A Multimodal AI Approach. Sensors.

[23] Muthulakshmi M, Kavitha G (2019). Deep CNN with LM learning based myocardial ischemia detection in cardiac magnetic resonance images.

[24] Cho GW, Sayed S, DCosta Z, Karlsberg DW, Karlsberg RP (2025). First comparison between artificial intelligence-guided coronary computed tomography angiography versus single-photon emission computed tomography testing for ischemia in clinical practice. Coron Artery Dis.

[25] Gonciar D, Berciu AG, Dulf EH, Orzan RI, Mocan T, Danku AE (2024). Computer-Assisted Algorithm for Quantification of Fibrosis by Native Cardiac CT A Pilot Study. J Clin Med.

[26] Curiale AH, Cabrera F, Jimenez P, Medus J, Mato G, Calandrelli ME (2022). Detection of Fibrosis in Cine Magnetic Resonance Images Using Artificial Intelligence. Techniques. arXiv.

[27] Campese S, Agostini F, Sciarretta T, Pizzi M, Cipriani A, Zanetti M (2022). Myocardial fibrosis detection using kernel methods preliminary results from a cardiac magnetic resonance study. Eur Heart J Cardiovasc. Imaging.

[28] Michalski B, Skonieczka S, Strzelecki M, Simiera M, Kupczynska K, Szymczyk E (2024). The use of artificial intelligence for predicting postinfarction myocardial viability in echocardiographic images. Cardiol J.

[29] Kumari SV, Lucas BR, Anitha C, Sangeetha SB, Santhi P, Raja RA (2025). Dense Residual Network-Powered Early Detection of Cardiovascular Diseases Using Multimodal Medical Imaging. J Neonatal Surg.

[30] Patel KK, Kanodia A, Kumar B, Gupta R (2024). Multi-Modal Data Fusion Based Cardiac Disease Prediction using Late Fusion and 2D CNN Architectures.

[31] Shen L, Zhang X, Huang S, Wu B, Li J (2023). A diagnostic method for cardiomyopathy based on multimodal data. Biomed Tech (Berl).

[32] Mota D, Filho FNB, Sousa EB, Chagas RA, Sassaki EM, Candoti MW (2024). Comparative analysis of classical artificial intelligence and deep learning models for atrial fibrillation detection in electrocardiograms. Europace.

[33] Jin Y, Ko B, Chang W, Choi KH, Lee KH (2025). Explainable paroxysmal atrial fibrillation diagnosis using an artificial intelligence-enabled electrocardiogram. Korean J Intern Med.

[34] Wu C, Hwang M, Huang TH, Chen YMJ, Chang YJ, Ho TH (2021). Application of artificial intelligence ensemble learning model in early prediction of atrial fibrillation. BMC Bioinformatics.

[35] Taniguchi H, Takata T, Takechi M, Furukawa A, Iwasawa J, Kawamura A (2021). Explainable Artificial Intelligence Model for Diagnosis of Atrial Fibrillation Using Holter Electrocardiogram Waveforms. Int Heart J.

[36] Zhong D, Huang L, Jin S, An Y, Zhu S, Li J (2023). Automatic electrocardiograph diagnosis of myocardial ischemia with support vector machine. Digit Med.

[37] Zhao X, Zhang J, Gong Y, Xu L, Liu H, Wei S (2022). Reliable Detection of Myocardial Ischemia Using Machine Learning Based on Temporal-Spatial Characteristics of Electrocardiogram and Vectorcardiogram. Front Physiol.

[38] Braun T, Spiliopoulos S, Veltman C, Hergesell V, Passow A, Tenderich G (2020). Detection of myocardial ischemia due to clinically asymptomatic coronary artery stenosis at rest using supervised artificial intelligence-enabled vectorcardiography - A five-fold cross validation of accuracy. J Electrocardiol.

[39] Jiang Z, Song W, Yan Y, Li A, Shen Y, Lu S (2024). Automated valvular heart disease detection using heart sound with a deep learning algorithm. Int J Cardiol Heart Vasc.

[40] Ma S, Chen J, Ho JWK (2024). An edge-device-compatible algorithm for valvular heart diseases screening using phonocardiogram signals with a lightweight convolutional neural network and self-supervised learning. Comput Methods Programs Biomed.

[41] Jamil S, Roy AM (2023). An efficient and robust Phonocardiography (PCG)-based Valvular Heart Diseases (VHD) detection framework using Vision Transformer (ViT). Comput Biol Med.

[42] Rancier MA, Israel I, Monickam V, Prince J, Verschoore B, Currie C (2023). Abstract 13244: Real World Evaluation of an Artificial Intelligence Enabled Digital Stethoscope for Detecting Undiagnosed Valvular Heart Disease in Primary Care. Circulation.

[43] Begic E, Gurbeta Pokvic L, Begic Z, Begic N, Dedic M, Mrsic D (2021). From Heart Murmur to Echocardiography - Congenital Heart Defects Diagnostics Using Machine-Learning Algorithms. Psychiatr Danub.

[44] Xu C, Li X, Zhang X, Wu R, Zhou Y, Zhao Q (2023). Cardiac murmur grading and risk analysis of cardiac diseases based on adaptable heterogeneous-modality multi-task learning. Health Inf Sci Syst.

[45] Ozcan F (2024). Rapid detection and interpretation of heart murmurs using phonocardiograms, transfer learning and explainable artificial intelligence. Health Inf Sci Syst.

[46] Tonni SI, Alam MA, Sohel A, Khan C, MdKR Onik, Pranto IH (2025). Predictive Modeling of Cardiovascular Disease Using Machine Learning: A Comparative Analysis.

[47] Sembina G, Aitim A, Shaizat M (2022). Machine Learning Algorithms for Predicting and Preventive Diagnosis of Cardiovascular Disease.

[48] Kumar HS, Sirapu T, T S, Penubaka KKR, Reddy PRKK, Supriya PL (2025). Investigating the Prediction of Cardiovascular Diseases using Different Machine Learning Methods.

[49] Ekong A (2023). Evaluation of Machine Learning Techniques Towards Early Detection of Cardiovascular Diseases. American Journal of Artificial Intelligence.

